# Molecular characterization of the first saltwater crocodilepox virus genome sequences from the world’s largest living member of the *Crocodylia*

**DOI:** 10.1038/s41598-018-23955-6

**Published:** 2018-04-04

**Authors:** Subir Sarker, Sally R. Isberg, Natalie L. Milic, Peter Lock, Karla J. Helbig

**Affiliations:** 1 Department of Physiology, Anatomy and Microbiology, School of Life Sciences, La Trobe University, Bundoora, VIC 3086 Australia; 2 Centre for Crocodile Research, Noonamah, NT Australia; 3 School of Psychological and Clinical Sciences, Charles Darwin University, Darwin, NT Australia; 4 La Trobe Institute for Molecular Science, La Trobe University, Bundoora, VIC Australia

## Abstract

Crocodilepox virus is a large dsDNA virus belonging to the genus *Crocodylidpoxvirus*, which infects a wide range of host species in the order *Crocodylia* worldwide. Here, we present genome sequences for a novel saltwater crocodilepox virus, with two subtypes (SwCRV-1 and -2), isolated from the Australian saltwater crocodile. Affected belly skins of juvenile saltwater crocodiles were used to sequence complete viral genomes, and perform electron microscopic analysis that visualized immature and mature virions. Analysis of the SwCRV genomes showed a high degree of sequence similarity to CRV (84.53% and 83.70%, respectively), with the novel SwCRV-1 and -2 complete genome sequences missing 5 and 6 genes respectively when compared to CRV, but containing 45 and 44 predicted unique genes. Similar to CRV, SwCRV also lacks the genes involved in virulence and host range, however, considering the presence of numerous hypothetical and or unique genes in the SwCRV genomes, it is completely reasonable that the genes encoding these functions are present but not recognized. Phylogenetic analysis suggested a monophyletic relationship between SwCRV and CRV, however, SwCRV is quite distinct from other chordopoxvirus genomes. These are the first SwCRV complete genome sequences isolated from saltwater crocodile skin lesions.

## Introduction

The crocodilepox virus belongs to the genus *Crocodylidpoxvirus*, a member of the subfamily *Chordopoxvirinae* in the family *Poxviridae*. Whilst currently only represented by *Nile crocodilepox virus* (CRV), isolated from Nile crocodiles (*Crocodylus niloticus*), poxvirus-like lesions have been identified on many of the *Crocodylia* order worldwide^1–6^. However, relatively little is known about the origins, worldwide host distribution and genetic diversity of this class of viruses. Morphologically, the Nile crocodilepox virus (CRV) and other unclassified crocodilian poxvirus virions are similar to orthopoxvirus virions, demonstrating a brick-like shape with rounded corners and having a dumbbell-shaped central core and lateral bodies^1,3,7^. However, they also display the regular, crisscross surface structure pattern characteristic of parapoxvirus virions^7–11^.

Poxviruses have been shown to infect the crocodilians as well as other reptiles, including Hermann’s tortoise, wild flap-necked chameleon, and lizards around the world^12^. The first report of poxvirus-associated disease in a reptile was in captive caimans (*Caiman crocodilus*) in the USA, and the affected animals have been shown to develop gray-white skin lesions on various parts of the body^9^. In Nile crocodiles (*C*. *niloticus*), poxvirus infections have been associated with high morbidity but low mortality, and the affected skin demonstrates brownish wart-like lesions that can occur over the entire body^5,13^.

Saltwater crocodiles (*Crocodylus porosus*) also present with poxviral lesions and the virus is considered a significant pathogen causing substantial economic loss for producers in Australia^1,3–5,14^. *C*. *porosus* are farmed mainly to produce high-quality leather for the international leather market and any disease that results in downgrading of hides causes significant financial loss^2,3^. A recent study by Moore *et al*.^3^ confirmed that there was no breach in the basement membrane of poxvirus infected saltwater crocodile skins using histopathological examination on different phases of poxvirus lesion progression. This study also postulated that allowing enough time for poxvirus infection to resolve might have no detrimental effect on skin quality if there are no obvious poxvirus lesions developing in the interim. In contrast to this study, Huchzermeyer *et al*.^15^ showed that there was a potential connection between poxvirus infection and deep lesions that remained as large depressed foci on the tanned skin of *C*. *niloticus*. In addition, the raised nodules (6–8 mm)^7,13^, and brown discolouration seen on live animal skins of *C*. *niloticus*^5^.

Although crocodile poxvirus has evolved to infect the species within the order *Crocodylia* worldwide, to date only one crocodile poxvirus genome has been published; a Nile crocodilepox virus (CRV)^1^. Excepting the recent study by Moore *et al*.^3^, who have demonstrated the genetic evidence of poxvirus in saltwater crocodile using a PCR targeted to amplify partial sequences, there is no other study characterizing the complete genome of saltwater crocodilepox virus (SwCRV). Therefore, the aim of the present study was to identify and characterize genetically and microscopically of the SwCRV genome sequences and virion morphology respectively that is associated with clinical disease in Australian saltwater crocodiles sourced from the Darwin Crocodile Farm, Northern Territory in 2017. To the best of our knowledge, this is the first report of complete poxvirus genome sequences from the saltwater crocodile.

## Results

### Identification of poxvirus infection in farmed saltwater crocodiles (*C. porosus*)

**Figure 1:**
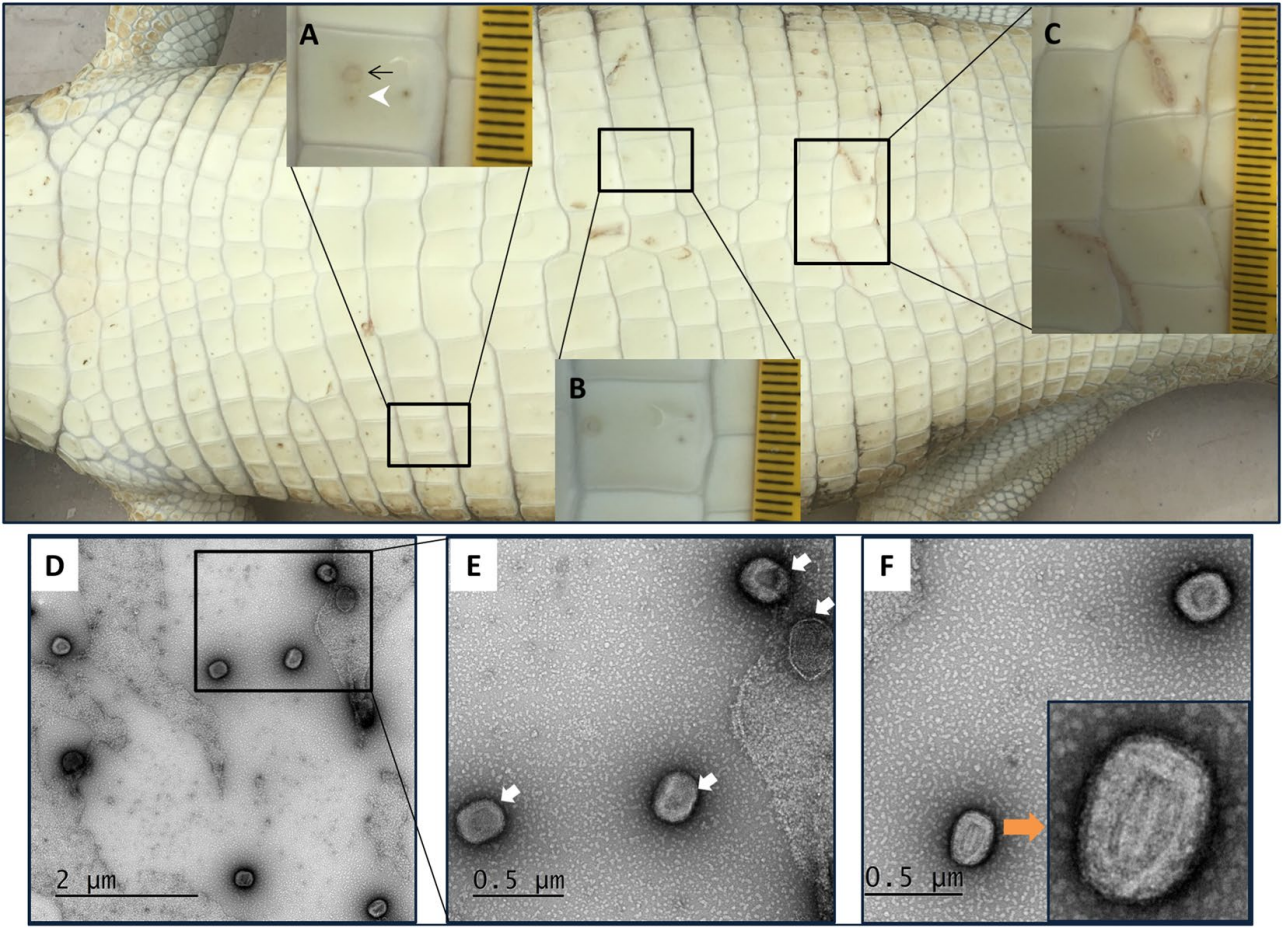
Macroscopic (top panel) and transmission electron microscopic (bottom panel) analysis of saltwater crocodile tissues infected with poxvirus. Belly skin of juvenile saltwater crocodile showing poxvirus lesions as defined by Moore *et al*.^3^ (**A**) has an early active (black arrow) and active (white arrowhead) poxvirus lesion in the mid-scale region, (**B**) has an active lesion on the upper scale margin, and (**C**) shows poxvirus lesions along two linear blemishes ranging from the active to expulsion stages. Different stages of virus maturation (**D**–**F**) including immature virion (IV) (**E**, white arrow), and intracellular mature virion (IMV) (**F**, orange arrow) were imaged by transmission electron microscopy.

Affected belly skin of a juvenile saltwater crocodile demonstrated an early active poxvirus lesion in the mid-scale region (Fig. 1A), an active lesion on the upper scale margin (Fig. 1B), and two linear blemishes ranging from the active to expulsion stages (Fig. 1C) of poxvirus lesions as defined by Moore *et al*.^3^. Transmission electron microscopy (TEM) analysis of negatively stained exudate sourced from two different juvenile saltwater crocodiles was performed. Two stages of viral enveloped including brick-shaped virus particles (Fig. 1E and F) indicating active poxvirus infection in these crocodiles were identified by TEM. According to Harrison *et al*.^16^, two different stages of virus particles can be observed: the immature virion (IV) (Fig. 1E, white arrow) and an intracellular mature virion (IMV) (Fig. 1E, orange arrow). Morphologically, the immature virions were brick shaped with rounded corners, whereas the intracellular mature virion had a dumbbell-shaped central core with lateral bodies. The virions had a length of approximately 200 to 250 nm and a width of 120 to 150 nm. To further confirm the presence of poxvirus, a PCR targeting a conserved region of the RNA polymerase subunit gene was performed^17^.

### Genome structure and analysis of SwCRV

**Figure 2:**
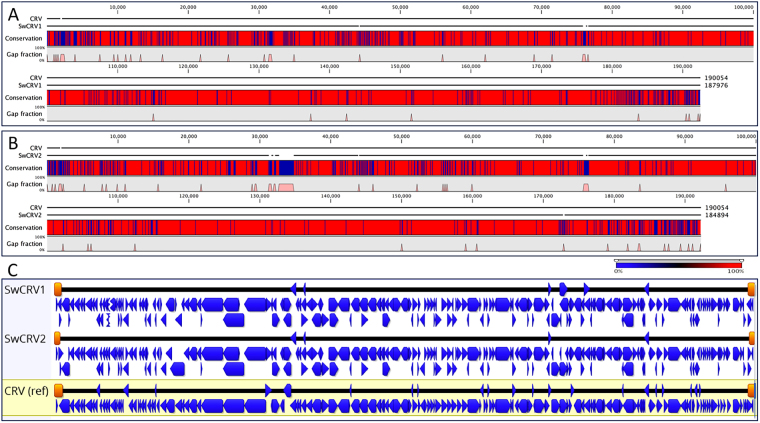
Comparative genome architectures of the SwCRV-1 and -2. (**A**) Sequence alignment of saltwater crocodilepox virus subtype 1 (SwCRV-1, GenBank accession number MG450915) to the reference Nile crocodilepox virus (CRV, DQ356948) genome. (**B**) Sequence alignment of saltwater crocodilepox virus subtype 2 (SwCRV-2, GenBank accession number MG450916) to the reference Nile crocodilepox virus (CRV, DQ356948) genome. The alignment was performed using the global alignment program contained in CLC Genomic Workbench (tool for Classical sequence analysis). The middle graphs in A and B represent the sequence conservation between the aligned SwCRV and CRV sequences at a given coordinate on the base sequence. The bottom graphs in A and B represent the gap fractions which are mostly for insertion and deletion between two representative viral genomes. (**C**) A sequence alignment using MAFFT in Geneious (version 10.2.3), and comparative ORF map of SwCRV and CRV. Protein coding ORFs, with blue arrows depicting the direction of transcription, whereas the orange blocks depicted Inverted Terminal Repeats (ITRs), respectively.

Poxvirus genomic material was isolated from two separate poxvirus lesions on two separate crocodiles both from Darwin Crocodile Farm. The assembled saltwater crocodilepox virus subtypes 1 and 2 (SwCRV-1 and -2) complete genomes were linear double-stranded DNA molecules of 187,976 and 184,894 bp, respectively. Nucleotide composition averaged over 62% G + C content for each of the two isolates analysed here (Fig. 2A), which is relatively high compared to other chordopoxviruses (ChPVs). Like Nile crocodilepox virus (CRV), SwCRV genomes contained a large central coding region bounded by two identical inverted terminal repeat (ITR) regions. The assembled ITRs of SwCRV-1 and -2 contained 1,700 and 1,254 bp, respectively. Large variations in the size of ITRs were detected between SwCRV-2 and CRV genomes, and this variation has commonly been found in other ChPVs^18,19^. Importantly, each of the inverted repeats constitutes arrays of direct repeats, and five tandem repeats detected within each ITR region of SwCRV-1, which consisted of 18, 12, and three 6 bp repeat units that shared approximately 88–100% nucleotide identity with each other. Whereas, ITRs of SwCRV-2 comprised four tandem repeats within each ITR region consisting of an 18, two 12, and one 6 bp repeat unit and sharing approximately 88–91% nucleotide identity with each other. These direct repeat arrays are smaller in size than those detected previously in other ChPVs, however we cannot rule out the possibility that they extend beyond the sequenced portion of the genome.

Further analysis of ITRs regions of SwCRV, detected a putative concatemer resolution motif (Supplementary Figure S1) in the very terminus of each ITR, and this motif has been found to be essential for the resolution of concatemeric DNA molecules during Vaccinia virus replication^20^. This motif spans nucleotides 126 to 145 and 79–98 (SwCRV-1 and -2, respectively) of each terminal nucleotide of the SwCRV two-DNA strand region, which is consistent with the previous reports in the case of CRV and other members of the ChPVs^1,21^. The concatemer resolution motifs of SwCRV were 100% similar to CRV, and consistently these motifs have been detected within the 150-bp terminal-most regions of the two DNA strands of ChPV members^1,21^. Furthermore, a hairpin loop-like secondary structure has been predicted in the terminal region of the SwCRV genome, which is consistent with previous reports^21,22^, however, its functionality needs to be assessed further.

Despite the high G+C content of SwCRV and the paucity of stop codons, 218 and 215 methionine-initiated ORFs encoding proteins of 50 to 1894 amino acids (aa) in length were observed in SwCRV-1 and -2, respectively, and were numbered from left to right (Fig. 2B, and Supplementary Table S1). Comparison of the predicted ORF protein sequences to the non-redundant protein sequence database at the National Center for Biotechnology Information (NIH, Bethesda, MD) using BLASTP identified homologs with significant protein sequence similarity (E value ≤ e-4) for 173 and 171 genes in SwCRV-1 and -2, respectively (Supplementary Table S1). Interestingly, there were 45 and 44 predicted protein coding genes in SwCRV-1 and -2, respectively, that were not present in any other poxvirus, nor did they match any sequences in the NR protein database using BLASTP; these unique ORFs encoded proteins of 50 to 876 aa in length (Supplementary Table S1). Among the predicted protein coding genes without detectable homologs, three contained predicted transmembrane segments detected by HHpred (Supplementary Table S1).

Among the predicted protein-coding genes of the SwCRV-1 and -2 genomes, respectively 173 and 171 predicted gene were homologs to other ChPV gene products (Supplementary Table S1). Among these conserved chordopoxvirus gene products, the highest number of protein-coding genes in SwCRV-1 and -2 genomes (168 and 167, respectively) demonstrated homology to the Nile crocodilepox virus. In contrast to CRV, the genomes of SwCRV-1 and CRV-2 included two additional genes encoding virion protein (SwCRV-1, ORF145 and SwCRV-2, ORF146) and early transcription factor VETFs (SwCRV-1, ORF147 and SwCRV-2, ORF144) which were identified as homologs of gene products of the Orf parapox virus in sheep (Supplementary Table S1). Furthermore, the SwCRV-1 genome included an insertion sequence encoding an uncharacterized protein (ORF156), also from the Orf parapox virus in sheep (Supplementary Table S1). As expected from the higher percent nucleotide identity between SwCRV and CRV (84.53 and 83.70%, respectively), SwCRV-1 and -2 were found to be considerably closer to CRV. In comparison to CRV, five ORFs with unknown functions (CRV024, CRV029, CRV156, CRV171 and CRV172) were commonly missing in both the SwCRV-1 and -2 genomes, and a further ORF of CRV (CRV035) was missing in SwCRV-2. A further 2 genes (CRV-ORF41 and ORF58) were significantly fragmented in both of the SwCRV genomes. All conserved genes of SwCRV-1 and -2 showed the highest sequence similarity to the orthologs from CRV and orthopoxvirus, and these observations imply that the conserved SwCRV-1 and -2 genes share an evolutionary history (Supplementary Table S1) with them. Overall, the central genomic core region of the SwCRV-1 and -2 contained homologues of conserved poxvirus genes involved in basic replicative mechanisms (RNA transcription and modification, and DNA replication), in virion structure, and in morphogenesis of intracellular mature, intracellular enveloped, and extracellular enveloped virions (IMV, IEV and EEV, respectively). The terminal regions being highly divergent, containing most of the predicted unique genes (Fig. 2B and Supplementary Table S1); which is consistent with other ChPVs^23,24^.

### Comparison between SwCRV-1 and -2 genomic structures

At the genomic level, SwCRV-1 and -2 genomes shared 97.80% nucleotide identity, and contain 205 genes with the same relative order and orientation, of which 43 were unique to SwCRV. Recent studies by Sarker *et al*.^25^ postulated that a 1% difference between genomes corresponds to approximately 10 mutations in an average sized gene any of which could significantly affects gene function if an early STOP codon is introduced to the gene sequence. Likewise, small changes within the promoter and enhancer regions of genes can significantly alter gene expression levels. Comparison of the two SwCRV genomes showed that three ORFs (SwCRV1–044, 045 and 156) with unknown functions were missing in SwCRV-2 (Supplementary Table S1). Furthermore, there were several occurrences of gene translocations, including the position of genes, observed between the two SwCRV genomes (highlighted as grey shading in Supplementary Table S1). Collectively these points of difference support inclusion of SwCRV-1 and SwCRV-2 as separate subtypes within the same species.

### SwCRV proteins of special interest

#### F-box proteins

F-box proteins are critical components for catalysing the ubiquitination of proteins, and serve as substrate-specific/recognition subunits in many critical cellular functions^26–28^. However, the function of F-box proteins found in poxviruses is not well understood. As with CRV, SwCRV also contains 9 genes encoding F-box domains (Supplementary Table S1), which comprise the largest gene family within the ChPVs. However, the F-box proteins of SwCRV were relatively diverse in comparison to the homologous proteins encoded by CRV. The amino acid identities of encoded F-box genes between SwCRV and CRV ranges from 76.6 to 96.9%, with the highest diversity being demonstrated in ORF211 and ORF208 for the SwCRV-1 and -2, respectively. Seven of the SwCRV F-box proteins were located in tandem in the left-terminal genomic region, and ranged in size from 189 to 288 amino acids in length. Whereas, SwCRV1-ORF211 and SwCRV2-ORF208 encoded F-box proteins were located in the right-terminal genomic region, and showed significant divergence with respect to the homologous CRV F-box proteins (Supplementary Table S1).

### GyrB-like ATPase domain gene family

Both SwCRV-1 and -2 contained 7 genes (SwCRV1-ORF109, 110, 112, 114, 116, 117, 119, and SwCRV2-ORF107, 108, 110, 112, 114, 115, 117, respectively) encoding DNA gyrase B subunit (GyrB)-like ATPase domain that shared approximately 11 to 31% amino acid identity with each other (Supplementary Table S1). The SwCRV GyRB-like ATPase domain gene family shared significant protein homology to the CRV GyrB-like ATPase domain of type II DNA topoisomerases (topo II), with amino acid identities ranging from 81 to 95%. Similar to CRV^1^, SwCRV GyrB-like ATPase domain ORFs were tandemly arranged in the central genomic location. However, arrangement of the repeats does not share co-linearity with other ChPVs (Supplementary Table S1). In contrast to other ChPVs, the GyrB-like ATPase domain gene family was commonly found in the CRV genome, along with the newly sequenced SwCRV genomes from the present novel host species (*C*. *porosus*). This suggests that GyrB-like ATPase domain genes family is unique for the genus *crocodylipoxvirus*.

### B22R-like proteins

B22R-like genes are the largest known poxvirus genes that encode proteins of unknown function, but which are predicted to contain transmembrane domains^1^. SwCRV-1 and -2 contained four copies of the B22R gene (SwCRV1-ORF51–53, 55, and SwCRV2-ORF49–51, 53, respectively), that shared between 67 to 86% amino acid identity to the CRV B22R genes (Supplementary Table S1). In contrast to CRV, SwCRV1- and -2 contained an additional copy of a predicted B22R-like gene (SwCRV1–052 and SwCRV2–050, respectively), which also shared 80% protein homolog to CRV041. Comparable to CRV and avipoxviruses, SwCRV B22R-like genes were located in a genomic region, which was distinct from mammalian ChPV B22R genes homologues (Supplementary Table S1). Importantly, one of the predicted B22R-protein coding genes in SwCRV-1 and -2 (SwCRV1–053 and SwCRV2–051, respectively) encodes a product that is significantly shorter in length (1385 AA) than the encoded product of the homologous gene in CRV (1874 AA), and demonstrated a low-level of protein similarity (>67%) within the B22R-like gene family.

### Evolutionary relationships of SwCRV

**Figure 3:**
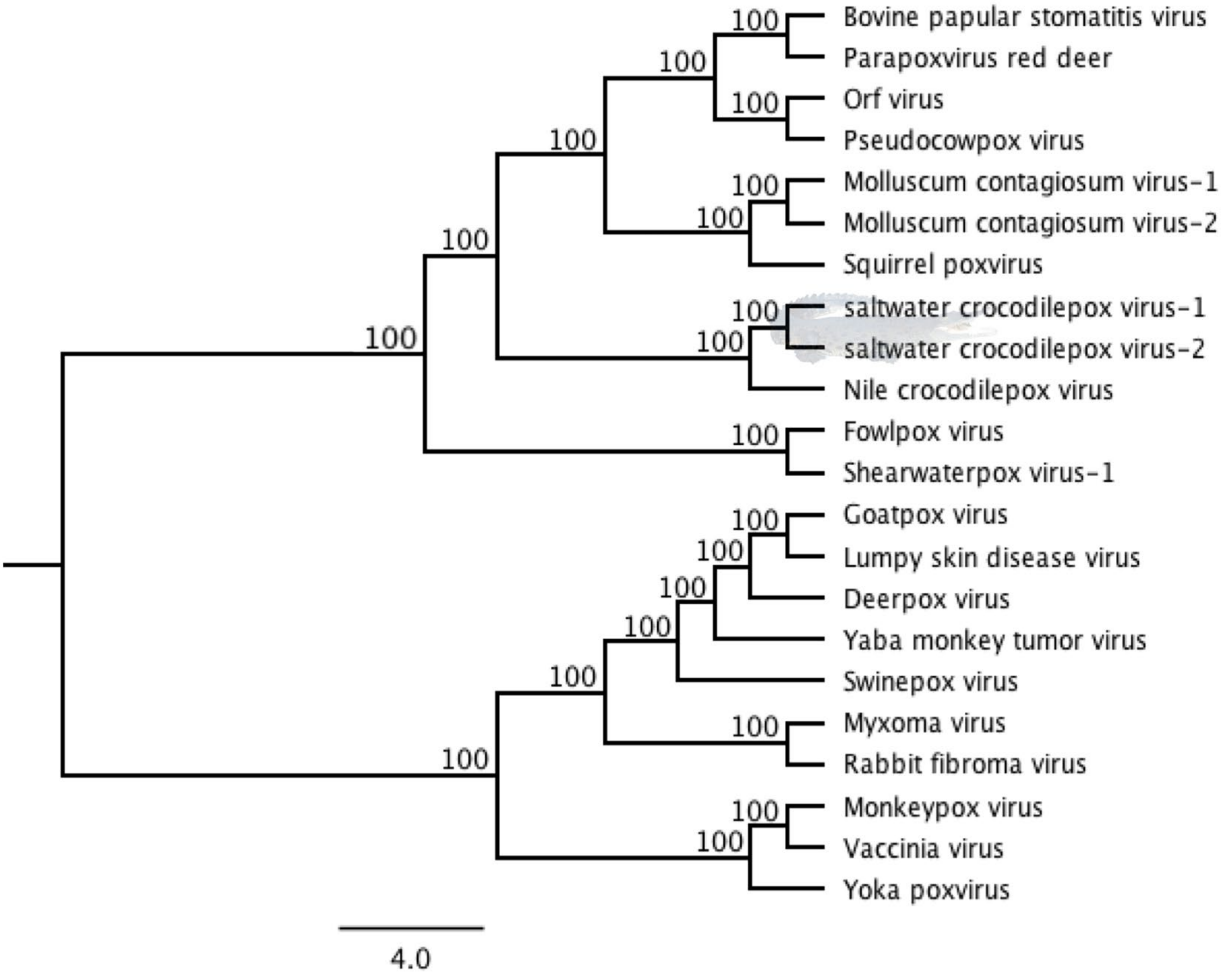
Phylogenetic tree among selected complete genome sequences of poxviruses. The ML tree was constructed from a multiple-nucleotide alignment from the selected complete genome sequences of poxviruses. The numbers on the left show bootstrap values as percentages, and the clade consisted with SwCRV was highlighted using saltwater crocodile shading (taken and supplied by author S.R.I.). The GenBank accession details for poxviruses were used: CRV (Nile crocodilepox virus, DQ356948); MOCV1 (Molluscum contagiosum virus subtype 1, MCU60315); MOCV2 (Molluscum contagiosum virus subtype 2, KY040274); SQPV (Squirrel poxvirus, HE601899); BPSV (Bovine papular stomatitis virus, KM875470); PPRD (Parapoxvirus red deer, KM502564); ORFV (Orf virus, DQ184476); PCPV (Pseudocowpox virus, GQ329670); SwCRV-1 (saltwater crocodilepox virus subtype 1, MG450915); SwCRV-2 (saltwater crocodilepox virus subtype 2, MG450916); DPV (Deerpox virus, AY689436); GPV (Goatpox virus, KC951854); MYXV (Myxoma virus, KP723391); RFV (Rabbit fibroma virus, AF170722); LSDV (Lumpy skin disease virus, NC_003027); FWPV (Fowlpox virus, AF198100); SWPV-1 (Shearwaterpox virus-1, KX857216); YMTV (Yaba monkey tumor virus, NC_005179); MPXV (Monkeypox virus, JX878407); VACV (Vaccinia virus, AY678275); YKPV (Yoka poxvirus, HQ849551); SWPV (Swinepox virus, NC_003389).

Considering the fact that the saltwater crocodilepox virus subtypes 1 and 2 (SwCRV-1 and -2) were genomically most closely related to CRV, multiple-nucleotide alignments from selected complete poxvirus genome sequences were used to construct a ML phylogenetic tree and calculate the distance matrix. The unrooted phylogenetic tree (Fig. 3) derived from these complete genome sequence alignments confirmed that these two SwCRV subtypes, isolated from saltwater crocodiles, were mostly related to CRV, and revealed the highest closest homology to the CRV sequence (84.53% and 83.70% sequence identity between CRV and SwCRV-1 and -2, respectively, Fig. 3). A higher distance between the SwCRV and other selected poxvirus genome sequences was observed by highlighting the low level of sequence identity (22.32 to 33.07%).

**Figure 4:**
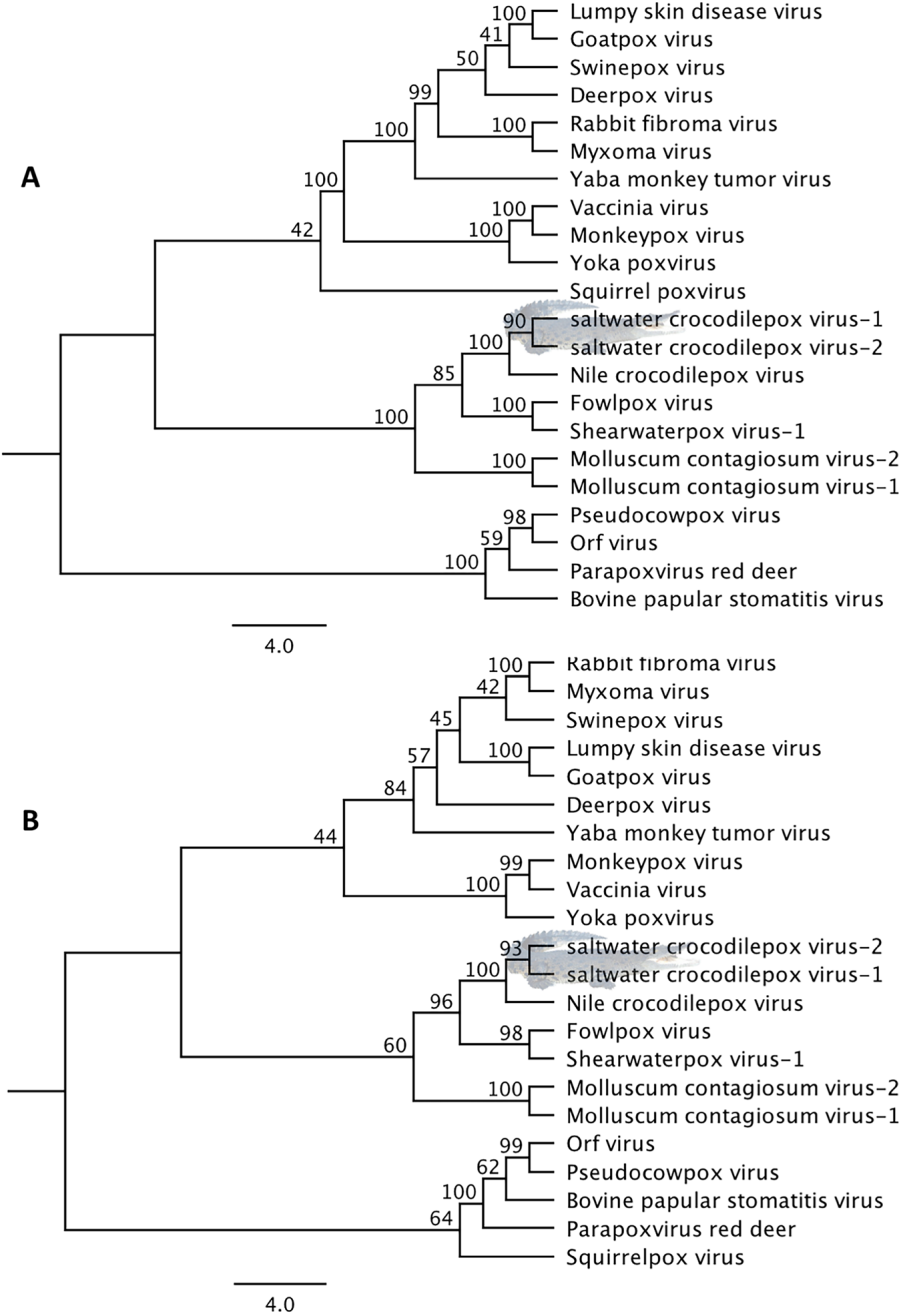
Phylogenetic tree and pairwise comparison of the DNA polymerase and DNA topoisomerase genes. The ML tress were constructed from the protein sequences of selected poxviruses using DNA polymerase genes (**A**), and DNA topoisomerase genes (**B**). The numbers on the left show bootstrap values as percentages, and SwCRV clade was highlighted using saltwater crocodile shading (taken and supplied by author S.R.I.). The abbreviations for poxviruses were used: CRV (Nile crocodilepox virus); MOCV1 (Molluscum contagiosum virus subtype 1); MOCV2 (Molluscum contagiosum virus subtype 2); SQPV (Squirrel poxvirus); BPSV (Bovine papular stomatitis virus); PPRD (Parapoxvirus red deer); ORFV (Orf virus); PCPV (Pseudocowpox virus); SwCRV-1 (saltwater crocodilepox virus subtype 1); SwCRV-2 (saltwater crocodilepox virus subtype 2); DPV (Deerpox virus); GPV (Goatpox virus); MYXV (Myxoma virus); RFV (Rabbit fibroma virus); LSDV (Lumpy skin disease virus); FWPV (Fowlpox virus); SWPV-1 (Shearwaterpox virus-1); YMTV (Yaba monkey tumor virus); MPXV (Monkeypox virus); VACV (Vaccinia virus); YKPV (Yoka poxvirus); SWPV (Swinepox virus).

To better understand these evolutionary relationships, the complete coding sequences of the DNA polymerase and DNA Topoisomerase genes were utilised to assemble phylogenetic trees and calculate distance matrixes, as has been performed previously^24,29^. A robust clade support (100%) between the two SwCRV and CRV was shown using a phylogenetic tree analysis in combination with a pairwise amino acid comparison using the DNA polymerase gene (Fig. 4A), with the DNA polymerase gene orthologs of SwCRV demonstrating amino acid identities ranging between 43.76 and 94.24%, to other chordopoxviruses (Supplementary Figure S2A). Interestingly, we also observed a closer and well-resolved evolutionary relationship using the DNA topoisomerase coding sequences (Fig. 4B), which demonstrated a very strong clade support (100%) between the SwCRV’s and CRV (Fig. 4B), with the protein sequence of the DNA topoisomerase gene of SwCRV exhibiting the highest (>96% aa identity) similarity with CRV (Supplementary Figure S2B).

## Discussion

This paper fully characterises the genomes of the first saltwater crocodilepox virus subtypes 1 and 2 (SwCRV-1 and -2) directly from typical poxvirus lesions on juvenile *C*. *porosus* belly skins. Poxvirus infection in juvenile saltwater crocodile was first reported in 1992^30^ and a very recent study has confirmed the presence of poxvirus-like particles in the lesion by using electron microscopy, and via PCR amplification of the RNA polymerase subunit gene^3^. At this stage, no taxonomic classification has been granted for SwCRV by the International Committee on Taxonomy of Viruses (ICTV; https://talk.ictvonline.org/taxonomy/)^6^ due to the lack of genome sequence data and unclear evolutionary history with other members of the family *Poxviridae*. In this study, we determined two novel SwCRV subtypes 1 and 2 (SwCRV-1 and -2) complete genome sequences with predicted full-length coding regions including ITRs, and these SwCRV genomes should be considered as new species under the genus *Crocodylidpoxvirus*. We also further established the ancestry history of SwCRV with other closely related members of the *Chordopoxvirinae* subfamily.

The nucleotide sequences of SwCRV-1 and -2 are significantly different to the Nile crocodilepox virus (CRV) (>84% and >83% similarity, respectively) but had close similarity to each other (>97%). SwCRV-1 and SwCRV-2 both displayed genetic distance and a novel genome structure in comparison to CRV, including the absence of 5 and 6 genes respectively, and the significant fragmentation of a further 2 genes most likely to cause them to be non-functional. With the exception of three predicted protein coding genes which have been detected to contain transmembrane segments using HHpred, SwCRV-1 and -2 contained 45 and 44 predicted protein-coding genes respectively, which are not found in any other known proteins in the NIH database, and is overall sufficiently genetically different to be considered a separate virus species.

Similar to CRV, SwCRV also lacks the genes involved in virulence and host range, including those involving the interferon response, intracellular signalling, and host immune response modulation. Nonetheless, the novel SwCRV-1 and -2 complete genome sequences contained an additional 45 and 44 predicted ORFs, respectively with unknown functions, which are found to be unique at this time. It might be possible that these unique ORFs in SwCRV potentially have roles to modulate host immune response, which needs to be examined further. Recent studies in eukaryotes have revealed that the small ORFs could represent potential steps in gene, peptide and protein evolution^31,32^. For example, small ORFs (sORFs; < 100 amino acids) in *Saccharomyces cerevisiae* are essential for viability^33^, whereas, several peptides encoded by sORFs are involved in activating transcription factors related to epidermal morphogenesis in *Drosophila*^34^.

Like many other members of the *Poxviridae*, crocodile poxvirus encodes multiple proteins which may have functions related to ubiquitin ligase (E3) enzyme components of the ubiquitin proteolytic pathway. This pathway is a potent mechanism for these viruses to selectively target proteins to alter protein functions and/or sort specific protein targets to the proteasome for degradation. Although these proteins are known to be important in some poxvirus lifecycles, their exact function remains unknown^1,35–39^. Similar to CRV, SwCRV contains a number of proteins related to ubiquitination, including proteins containing F-box motifs, Really Interesting New Gene (RING) finger motifs and one homologue of anaphase-promoting complex (APC)/C subunit 11 (Apc11) (Supplementary Table S1). F-box-like motifs were also recently detected in poxvirus ankyrin repeat (ANK) proteins, however, clear compositional differences to typical F-box proteins raise questions regarding the classification and function of this motif ^38^. *In-vivo* studies on Orf virus demonstrate that poxviral F-box-like motif acts as a functional F-box, and it interacts dependently on the poxviral F-box-like motif and the adaptor subunit of the complex (SKP1)^38^. However, the function and significance of the F-box-like motif more broadly in crocodile poxviruses remains to be discovered.

The SwCRV genomes also encode for an APC11-like protein (79 amino acids), which is a member of the anaphase promoting complex/cyclosome, the largest known cellular ubiquitin ligase complex, which is involved in controlling cell-cycle regulation^40^. The swCRV APC-like protein is homologous to Nile crocodilepox virus CRV047, Molluscum contagiosum virus (MOCV) MC026L, parapox virus 014, and Squirrelpox virus 026L. Only a small number of poxviruses encode Apc11 homologues, a protein which demonstrates sequence similarities to the mammalian cellular APC/C subunit 11 (Apc11), which has evolved from a RING-type E3 ligase, and perhaps suggests that the ring box E3 subunits originated from a diverse host species^36,41^. One of the hallmark functions of the RING-H2 domain of cellular Apc11 proteins is to allow ubiquitination of protein substrates after a direct interaction with E2 enzymes to recruit it to the APC^42–44^. However, sequence analysis has demonstrated that the poxvirus APC11-homolouge proteins contain sequence mutations in the E2 ligase binding domain, rendering them unable to bind E2 ligases^39^. Both SwCRV-1 and SwCRV-2 display high similarity to the CRV protein CRV047 (92.7%, Supplementary Table S1), and also contain sequence mutations in the hypothesised E2 ligase binding domain of the APC11-like protein. The functionality of these APC11- homologues in other poxviruses is not well understood but recent studies looking at PACR function, the Orf virus APC11-like protein, demonstrated that expression of the viral protein led to cell cycle deregulation and acted as a negative regulator of APC function. Deletion of this gene from the Orf virus also led to reduced viral replication^41^. Thus, it has been hypothesised that poxvirus APC-11-homolouges may act as viral mimics of the mammalian APC11 gene, to negatively regulate cell cycle and promote viral replication.

Similar to other chordopoxviruses, SwCRV also contained four copies of the B22R gene that shared approximately between 67 to 86% amino acids identity to the CRV B22R gene. In contrast to CRV, both SwCRV genomes had an additional copy of the predicted B22R-like gene. B22R is a surface glycoprotein that is well conserved in the ChPVs genus and has only one possible homolog outside the poxvirus family, in cyprinid herpesvirus 3, member of the family *Alloherpesviridae*^45^. B22R is present in every chordopoxvirus genus except parapoxvirus, and is the largest known protein encoded by poxviruses. It is predicted to contain carboxyl-terminal transmembrane domains and cysteine residues which may mediate disulfide bond formation, however, its function is still unknown^45,46^.

With the exception of bacteria like *Treponema pallidum subsp*. *Pallidum*, *Thermotoga maritima*, *Bacillus subtilis* and *Halomonas variabilis*^47–50^, the GyrB-like ATPase domain has only been found in CRV and SwCRV genomes which makes them unique amongst the chordopoxviruses. GyrB-like ATPase domain is a member of the type-II topoisomerase family of the ATP-dependent enzymes that catalyze topological DNA rearrangement in bacteria^47^. In the case of Nile crocodilepox virus (CRV), it has been suggested that GyrB-like ATPase domain may have energy-dependent functions potentially involving novel host-virus interactions^1^, however, its actual functions in the poxviruses is still unknown and requires further examination.

The phylogenetic distribution of *Crocodylidpoxvirus* genomes indicates that Nile crocodiles, saltwater crocodiles and perhaps other *Crocodylia* species could be important hosts for *Crocodylidpoxvirus* dispersal around the globe. As shown in Fig. 3, it is reasonable to postulate that these viruses perhaps originated from a common ancestor that diverged from a CRV-like progenitor. The reservoir hosts of these crocodile poxviruses may be the specific crocodile species, and/or other captive or wild animal species that are in close propinquity with the farmed crocodile host species. Without further experimentation, we cannot trace the actual source of poxvirus infection in the saltwater crocodile. Similar to other poxviruses^25,51^, it will not be surprising if vectors such as mosquitoes are playing a part during the transmission of poxvirus within the saltwater crocodile population.

Examining the phylogenetic tree and distance matrix using DNA polymerase and DNA topoisomerase genes of the swCRV genomes and CRV, it was demonstrated that there was an obvious trend associated within the poxvirus species isolated under the genus *Crocodylidpoxvirus* (Fig. 4 and Supplementary Figure S2). Well-supported phylogenetic trees were constructed using both the DNA polymerase and DNA topoisomerase genes and they show that SwCRV is closely related to CRV. These results further conclude that SwCRV may be more closely linked at a conserved gene level than across the length of the genome, highlighting the importance of complete genome characterization in comparison to single gene phylogenies. However, given their genetic diversity and geographically discrete distributions, it is perhaps not surprising that the saltwater crocodile and the Nile crocodile species may be exposed to different poxvirus infections.

## Conclusions

These are the first crocodilepox virus genome sequences isolated from a poxvirus infection of the Australian saltwater crocodile. The novel complete genome sequences of SwCRV subtypes 1 and -2 (SwCRV-1 and -2) are significantly divergent, but most similar to the CRV. Together with the sequence similarities observed between SwCRV and other chordopoxviruses, this study concluded that the SwCRV 1 and -2 complete genome sequences described here are not closely related to any other chordopoxvirus complete genomes isolated from avian, reptilian or other natural host species. As such, they should be considered as different subtypes of the same species, tentatively named as saltwater crocodilepox virus subtype 1 and saltwater crocodilepox virus subtype 2. Similar to CRV, SwCRV also lacks genes predicted to involve host immune response, however, it is completely reasonable to hypothesise that the genes encoding these functions are present but not recognized. Therefore, further studies for understanding the functions of hypothetical and/or uncharacterized proteins will be important to uncover whether or not these proteins are playing any role in host immune response modulation.

## Materials and Methods

### Source of samples

Exudate from characteristic poxvirus lesions on the belly skin of juvenile saltwater crocodiles were collected from Darwin Crocodile Farm (Noonamah, Northern Territory, Australia), as described by Moore *et al*.^3^, Northern Territory, Australia; (Fig. 1). Animal sampling was performed to comply with approved guidelines set by the Australian Code of Practice for the Care and Use of Animals for Scientific Purposes (1997) and approved by the Charles Darwin University Animal Ethics Committee (A16005).

### DNA extraction, llumina library preparation and sequencing

Tissue from individual pox lesions was aseptically dissected and mechanically homogenized in lysis buffer using disposable tissue grinder pestles and transferred into a 1.5 mL microcentrifuge tube (Eppendorf). Virion enrichment and DNA extraction from the sample was performed according to the protocol described by Sarker *et al*.^25,52^. DNA libraries were prepared according to published protocol^53^ using the Illumina Nextera XT DNA Library Prep V3 Kit starting with one ng of total genomic DNA (gDNA) as measured by Qubit (Invitrogen) and sequenced on the Illumina MiSeq platform.

### Bioinformatics

Sequencing data was analysed according to a previously established pipeline^25,53^ and using CLC Genomics workbench 9.5.4. A total of 2,263,362 and 770,348 pairs of 301-bp reads for the SwCRV-1 and -2, were obtained respectively. Preliminary quality evaluation for all raw reads was generated, pre-processed to remove ambiguous base calls and poor quality reads, and trimmed as previously described^53^. Trimmed sequence reads were aligned to the Australian saltwater crocodile genome (*Crocodylus porosus*, GenBank accession number NW_017728899) to remove host DNA contamination, with a total of 4.77% reads being excluded from further analysis. Reads were assembled, and contigs with high confidence were chosen for downstream analysis as previously described^53^. BLASTN analysis of the resulting contigs confirmed the closest match to be Nile crocodilepox virus. The Nile crocodilepox virus genome sequence was used as a reference genome, with selected contigs being mapped, edited, ordered and orientated as previously described^53^. A draft genome was created from *de novo* contigs were used as a reference sequence to assemble against the trimmed reads to confirm further. This produced an average coverage of 1905.9 and 476.58 for the genome sequences of SwCRV-1 and -2, respectively (Supplementary Figure S3). The consensus genome sequences of SwCRV-1 and -2 were extracted from CLC Genomics workbench 9.5.4 using the following criteria (low coverage threshold 20, remove region with low coverage and join after removal). The final consensus genome sequences were 187,976 and 184,894 bp for SwCRV-1 and -2, respectively obtained from the Australian saltwater crocodile.

### Genome annotations

The SwCRV genomes were annotated using GATU^54^ and further verification of the predicted ORFs were performed as previously described^53^, with the exception that overlapping ORFs showing unique or potential poxviruses orthologs using BLASTP serach were annotated to investigate further whether or not the missing ChPVs conserved genes in SwCRV were present. Future in vitro experimental work will need to be performed to assess the potential functionality of overlapping reading frames. Additionally, HHpred using default parameters^55^ was used to search protein homologs for unique ORFs predicted in this study. The correct methionine start site, signs of truncation, correct stop codons, and validity of overlaps for SwCRV annotations were further examined using other poxvirus ortholog alignments. The tandem direct repeats were identified using the Tandem Repeats Finders^56^, and concatemer resolution motifs were analysed using Geneious (version 10.2.3). Hairpin loop-like secondary structure in the ITRs region of SwCRV were also predicted using tools available in Geneious (version 10.2.3)^57^.

### Analysis of genome sequences and generation of phylogenetic trees

Nucleotide sequences of poxviruses including a representative virus of each genus of the *Chordopoxvirinae* with newly sequenced SwCRV genomes were downloaded from GenBank: CRV (Nile crocodilepox virus, DQ356948); SQPV (Squirrel poxvirus, HE601899); MOCV1 (Molluscum contagiosum virus subtype 1, MCU60315); MOCV2 (Molluscum contagiosum virus subtype 2, KY040274); PPRD (Parapoxvirus red deer, KM502564); BPSV (Bovine papular stomatitis virus, KM875470); ORFV (Orf virus, DQ184476); PCPV (Pseudocowpox virus, GQ329670); SwCRV-1 (saltwater crocodilepox virus subtype 1, MG450915); SwCRV-2 (saltwater crocodilepox virus subtype 2, MG450916); DPV (Deerpox virus, AY689436); GPV (Goatpox virus, KC951854); RFV (Rabbit fibroma virus, AF170722); MYXV (Myxoma virus, KP723391); LSDV (Lumpy skin disease virus, NC_003027); FWPV (Fowlpox virus, AF198100); SWPV1 (Shearwaterpox virus-1, KX857216); YMTV (Yaba monkey tumor virus, NC_005179); MPXV (Monkeypox virus, JX878407); VACV (Vaccinia virus, AY678275); YKPV (Yoka poxvirus, HQ849551); SWPV (Swinepox virus, NC_003389).

Phylogenetic analysis and amino acid sequence alignment was performed as previously stated^53^. Except in the case of DNA polymerase and DNA topoisomerase, tree topology with 500 bootstrap re-samplings under LG substitution model was chosen to generate ML tree using tools available in Geneious (version 10.2.3).

### Transmission electron microscopy

Exudate removed from pox lesions was suspended 1:10 in phosphate buffered saline (PBS), homogenised, clarified and adsorbed onto 400-mesh copper EM grids, prior to staining and imaging on a JEOL JEM-2100 transmission electron microscope as previously described^53^.

### Data availability

The complete genome sequences of the SwCRV-1 and -2 were deposited in GenBank under the accession number MG450915 and MG450916, respectively. Raw sequencing data from this study has been deposited in the NCBI Sequence Read Achieve (SRA) under the accession number of SRP128935 (BioProject ID: PRJNA429047; BioSample accessions: SAMN08328748 and SAMN08328749) (http://www.ncbi.nlm.nih.gov/sra/).

## Electronic supplementary material

Supplementary Information
