# Supplementary material for: Molecular characterization of the first saltwater crocodilepox virus genome sequences from the world’s largest living member of the Crocodylia
Source: Sci Rep. 2018 Apr 4;8:5623. doi: 10.1038/s41598-018-23955-6 (PMC5884845; doi:10.1038/s41598-018-23955-6)
Supplement: Supplementary file 1 — Supplementary Information [file 41598_2018_23955_MOESM1_ESM.pdf]

Supplementary Material

Molecular characterization of the first saltwater crocodilepox virus genome sequences from the world's largest living member of the *Crocodylia*

Subir Sarker[1,*], Sally R Isberg[2,3], Natalie L Milic[3], Peter Lock[4], Karla J Helbig[1]

[1]Department of Physiology, Anatomy and Microbiology, School of Life Sciences, La Trobe University, Bundoora, VIC 3086, Australia. [2]Centre for Crocodile Research, Noonamah, NT, Australia. [3]School of Psychological and Clinical Sciences, Charles Darwin University, Darwin, NT, Australia. [4]La Trobe Institute for Molecular Science, La Trobe University, Bundoora, VIC, Australia

[*]**Author for correspondence:** Dr Subir Sarker, Department of Physiology, Anatomy and Microbiology, School of Life Sciences, La Trobe University, Bundoora, VIC 3086, Australia, email: s.sarker@latrobe.edu.au; phone: +61 3 9479 2317; fax: +61 3 9479 1222.

Supplementary Figure: S1

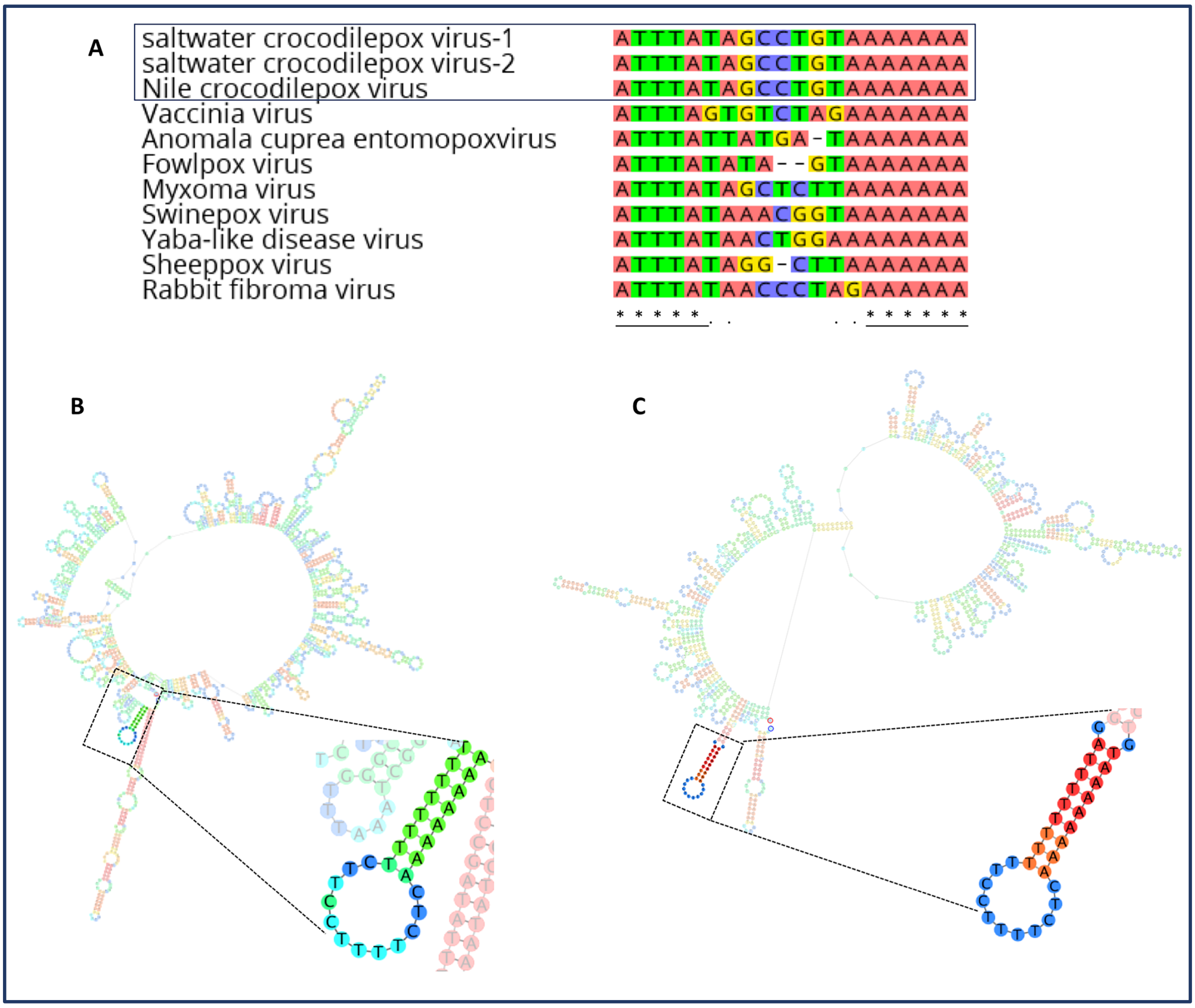

Supplementary Figure S1: Analysis of concatemer resolution motif and prediction of hairpin loop-like secondary structure of SwCRV genomes. (A) Alignment of the putative SwCRV concatemer resolution motif with those of other poxviruses. The motif is underlined. The analysis was performed by using Geneious software (version 10.2.3) and the resultant alignment was slightly modified manually. Asterisks and dots indicates 100% and >65% nucleotide identity, respectively, among the aligned sequences. Box highlights 100% similarity of concatemer resolution motif between CRV and SwCRV. GenBank accession details for poxviruses were used: shope (Rabbit) fibroma virus (AF170722); myxoma virus (AF170726); Yaba-like disease virus (AY386371); sheeppox virus (M28823); fowlpox virus (AJ581527); swinepox virus (AF410153); vaccinia virus (AY243312); crocodilepox virus (DQ356948); saltwater crocodilepox virus subtype 1 (MG450915); saltwater crocodilepox virus subtype 2 (MG450916). (**B**) Predicted hairpin loop-like secondary structure of SwCRV-1 formed by a 28 nucleotide (nt position 139-166; highlighted). (**C**) Predicted hairpin loop-like secondary structure of SwCRV-2 formed by a 31 nucleotide (nt position 90-120; highlighted). Colours of nucleotides represent base-pair probabilities (red=high, green=mid, blue=low).

Supplementary Figure: S2

A

		1	2	3	4	5	6	7	8	9	10	11	12	13	14	15	16	17	18	19	20	21	22
BPSV	1		0.14	0.13	0.14	0.63	0.62	0.63	0.63	0.63	0.62	0.64	0.59	0.58	0.60	0.53	0.64	0.65	0.76	0.77	0.75	0.75	0.75
ORFV	2	86.52		0.06	0.16	0.63	0.61	0.63	0.63	0.63	0.63	0.64	0.59	0.58	0.60	0.53	0.64	0.64	0.78	0.79	0.74	0.74	0.74
PCPV	3	88.01	94.05		0.16	0.62	0.61	0.63	0.63	0.63	0.62	0.63	0.59	0.58	0.60	0.52	0.64	0.64	0.76	0.78	0.74	0.74	0.74
PPRD	4	86.73	85.45	85.45		0.63	0.64	0.64	0.66	0.65	0.64	0.66	0.60	0.59	0.62	0.55	0.66	0.66	0.80	0.82	0.75	0.76	0.76
DPV	5	53.14	53.00	53.49	52.99		0.26	0.26	0.25	0.28	0.28	0.35	0.40	0.40	0.39	0.53	0.66	0.66	0.65	0.67	0.82	0.82	0.82
MYXV	6	53.69	54.14	54.14	52.85	77.15		0.08	0.29	0.29	0.30	0.36	0.42	0.41	0.40	0.54	0.67	0.67	0.67	0.67	0.80	0.80	0.80
RFV	7	53.25	53.50	53.50	52.81	76.36	92.25		0.30	0.32	0.33	0.38	0.42	0.42	0.42	0.55	0.70	0.70	0.69	0.69	0.83	0.82	0.82
SWPV	8	53.20	53.35	53.45	51.77	77.74	75.12	73.71		0.29	0.29	0.39	0.43	0.43	0.42	0.56	0.67	0.67	0.64	0.66	0.83	0.82	0.82
GPV	9	53.05	53.10	53.29	52.11	74.95	74.48	72.28	74.48		0.02	0.36	0.42	0.42	0.40	0.56	0.69	0.68	0.65	0.66	0.82	0.81	0.81
LSDV	10	53.34	53.20	53.49	52.40	75.15	73.99	72.08	74.98	98.12		0.36	0.42	0.42	0.40	0.55	0.69	0.69	0.66	0.67	0.82	0.81	0.81
YMTV	11	52.86	53.01	53.11	51.63	70.33	69.71	68.39	67.56	69.80	69.60		0.41	0.41	0.43	0.54	0.70	0.70	0.68	0.68	0.82	0.80	0.80
MPXV	12	55.22	55.17	55.07	54.57	66.44	65.91	65.48	64.95	65.32	65.51	66.37		0.01	0.25	0.52	0.65	0.65	0.69	0.67	0.77	0.77	0.77
VACV	13	55.81	55.76	55.67	55.26	66.93	66.20	65.58	65.15	65.51	65.81	66.37	98.71		0.25	0.51	0.64	0.64	0.69	0.67	0.77	0.78	0.78
YKPV	14	54.42	54.38	54.28	53.39	67.29	66.96	65.54	65.51	66.96	66.86	64.75	77.80	78.00		0.54	0.68	0.68	0.66	0.65	0.82	0.81	0.81
SQPV	15	57.69	57.94	58.63	56.90	58.39	58.13	57.59	56.50	56.48	56.97	58.09	59.07	59.47	57.78		0.53	0.53	0.70	0.72	0.72	0.71	0.71
MOCV1	16	52.32	52.47	52.66	51.67	52.02	51.29	50.15	51.39	50.44	50.35	49.95	52.52	52.72	50.64	58.31		0.01	0.67	0.68	0.66	0.68	0.68
MOCV2	17	52.22	52.47	52.56	51.57	51.92	51.29	50.05	51.68	50.64	50.54	50.05	52.52	52.72	50.74	57.82	98.61		0.67	0.67	0.66	0.68	0.68
FWPV	18	46.16	45.22	46.11	44.35	51.08	50.59	49.46	51.63	51.03	50.64	50.25	49.21	49.60	50.54	48.28	50.64	50.74		0.25	0.76	0.76	0.76
SWPV1	19	45.57	44.93	45.32	43.56	50.00	50.30	49.26	50.84	50.64	50.44	49.85	50.30	50.49	51.03	47.40	50.05	50.45	78.24		0.79	0.78	0.78
CRV	20	45.98	46.52	46.52	46.03	43.55	44.52	43.34	43.39	43.50	43.50	43.73	45.52	45.42	43.26	47.64	50.49	50.69	45.87	44.88		0.06	0.06
SwCRV1	21	46.27	46.52	46.61	45.84	43.84	44.62	43.63	43.59	44.19	44.09	44.42	45.42	45.32	43.76	47.84	49.51	49.61	46.27	45.37	94.24		0.00
SwCRV2	22	46.27	46.52	46.61	45.84	43.84	44.62	43.63	43.59	44.19	44.09	44.42	45.42	45.32	43.76	47.84	49.51	49.61	46.27	45.37	94.24	100.00	

B

		1	2	3	4	5	6	7	8	9	10	11	12	13	14	15	16	17	18	19	20	21	22
BPSV	1		0.14	0.12	0.16	0.64	0.65	0.65	0.69	0.70	0.71	0.71	0.55	0.55	0.60	0.51	0.74	0.79	0.67	0.68	0.81	0.80	0.80
ORFV	2	86.25		0.04	0.17	0.64	0.66	0.66	0.67	0.69	0.68	0.68	0.58	0.58	0.63	0.51	0.77	0.80	0.67	0.68	0.78	0.78	0.78
PCPV	3	87.81	95.91		0.17	0.65	0.67	0.67	0.69	0.69	0.70	0.69	0.58	0.58	0.63	0.52	0.76	0.82	0.68	0.69	0.80	0.79	0.79
PPRD	4	84.16	83.75	84.06		0.66	0.71	0.71	0.73	0.71	0.72	0.71	0.57	0.57	0.64	0.51	0.77	0.79	0.68	0.69	0.80	0.81	0.81
DPV	5	52.34	52.35	51.72	51.09		0.32	0.32	0.40	0.41	0.40	0.40	0.44	0.44	0.47	0.53	0.61	0.61	0.65	0.65	0.77	0.78	0.78
GPV	6	51.23	50.93	50.31	48.46	72.24		0.00	0.33	0.38	0.35	0.36	0.45	0.45	0.50	0.52	0.60	0.62	0.70	0.70	0.75	0.75	0.75
LSDV	7	51.23	50.93	50.31	48.46	72.24	99.68		0.33	0.38	0.35	0.36	0.44	0.44	0.50	0.52	0.60	0.62	0.70	0.70	0.75	0.75	0.75
SWPV	8	49.54	50.46	49.54	47.69	65.94	71.56	71.56		0.46	0.41	0.43	0.52	0.52	0.49	0.59	0.67	0.62	0.74	0.74	0.78	0.80	0.80
YMTV	9	49.07	49.69	49.69	48.76	65.82	68.45	68.45	62.50		0.44	0.44	0.47	0.46	0.50	0.51	0.64	0.60	0.77	0.77	0.79	0.79	0.79
MYXV	10	48.91	50.47	49.84	48.60	67.30	69.81	69.81	65.62	64.04		0.06	0.49	0.49	0.53	0.50	0.58	0.59	0.69	0.70	0.74	0.74	0.74
RFV	11	48.91	50.47	49.84	48.60	67.52	69.40	69.40	64.06	64.24	93.65		0.49	0.49	0.53	0.50	0.60	0.60	0.67	0.68	0.73	0.73	0.73
MPXV	12	57.01	55.49	55.49	55.76	64.13	63.72	64.04	59.06	62.86	61.08	61.27		0.00	0.27	0.48	0.60	0.61	0.63	0.63	0.73	0.74	0.74
VACV	13	57.01	55.49	55.49	55.76	64.13	63.72	64.04	59.06	63.17	61.08	61.27	99.68		0.27	0.48	0.60	0.61	0.63	0.63	0.73	0.74	0.74
YKPV	14	53.89	52.66	52.98	52.02	62.54	60.25	60.25	60.31	60.95	58.54	58.73	76.11	76.43		0.52	0.64	0.60	0.70	0.69	0.74	0.75	0.74
SQPV	15	59.13	59.19	58.88	59.13	58.49	59.43	59.43	55.14	59.94	59.87	60.38	61.51	61.51	58.99		0.67	0.67	0.56	0.57	0.73	0.73	0.73
FWPV	16	47.20	45.96	46.27	45.68	53.77	54.06	54.06	50.15	52.52	55.17	54.40	54.89	54.89	53.16	50.94		0.26	0.76	0.77	0.68	0.70	0.70
SWPV1	17	45.34	44.41	43.48	44.44	53.77	53.12	53.12	52.63	54.72	54.86	54.40	54.26	54.26	54.75	50.62	76.90		0.77	0.77	0.68	0.67	0.67
MOCV1	18	50.15	50.00	49.69	49.39	51.23	49.07	49.07	47.38	46.13	49.38	50.31	52.32	52.32	48.92	56.48	46.15	45.85		0.01	0.72	0.72	0.72
MOCV2	19	49.85	49.69	49.39	49.09	51.23	49.07	49.07	47.38	46.13	48.77	49.69	52.32	52.32	49.23	55.86	45.85	45.54	99.38		0.73	0.73	0.73
CRV	20	43.90	44.95	44.04	44.07	45.54	46.32	46.32	45.26	45.06	47.08	47.38	47.53	47.53	46.91	47.55	49.69	49.39	48.92	48.62		0.03	0.03
SwCRV1	21	44.21	45.26	44.65	43.47	44.92	46.32	46.32	44.34	45.06	46.77	47.08	46.91	46.91	46.60	47.24	48.77	50.00	48.92	48.62	96.60		0.00
SwCRV2	22	44.21	45.26	44.65	43.47	44.92	46.32	46.32	44.65	45.06	46.77	47.08	46.91	46.91	46.91	47.24	48.77	50.00	48.92	48.62	96.91	99.69	

min max

Supplementary Figure S2: Pairwise comparison of the viral DNA polymerase and DNA topoisomerase genes. (**A**) Pairwise comparison of protein sequences of the viral DNA polymerase genes of poxviruses. (**B**) Pairwise comparison of protein sequences of the viral DNA topoisomerase genes of poxviruses. Upper comparison gradient indicated the distance between two protein sequences, and lower comparison gradient indicated percentage identity between two protein sequences. The abbreviations for poxviruses were used: The abbreviations for poxviruses were used: CRV (Nile crocodilepox virus); MOCV1 (Molluscum contagiosum virus subtype 1); MOCV2 (Molluscum contagiosum virus subtype 2); SQPV (Squirrel poxvirus); BPSV (Bovine papular stomatitis virus); PPRD (Parapoxvirus red deer); ORFV (Orf virus); PCPV (Pseudocowpox virus); SwCRV-1 (saltwater crocodilepox virus subtype 1); SwCRV-2 (saltwater crocodilepox virus subtype 2); DPV (Deerpox virus); GPV (Goatpox virus); MYXV (Myxoma virus); RFV (Rabbit fibroma virus); LSDV (Lumpy skin disease virus); FWPV (Fowlpox virus); SWPV1 (Shearwaterpox virus-1); YMTV (Yaba monkey tumor virus); MPXV (Monkeypox virus); VACV (Vaccinia virus); YKPV (Yoka poxvirus); SWPV (Swinepox virus).

Supplementary Figure: S3

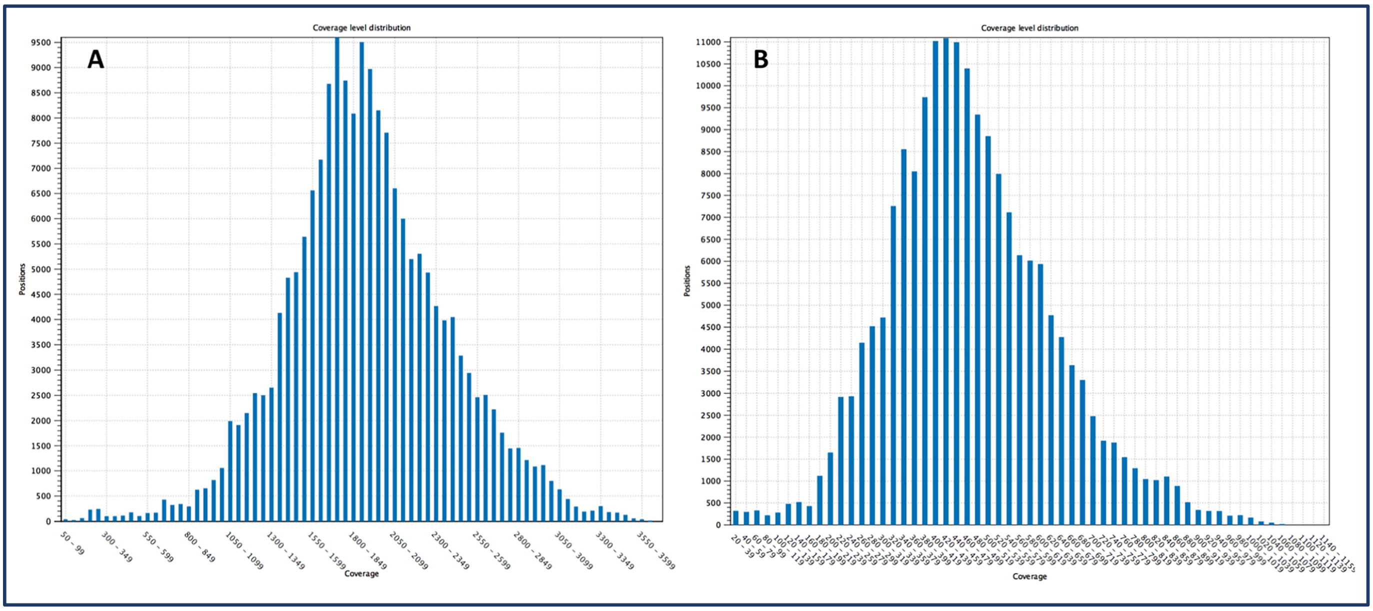

Supplementary Figure S3: Coverage level distribution for the genome sequences of SwCRV-1 (A) and SwCRV-2 (B). The horizontal axis represents the range of coverage, whereas the vertical axis represents the number of nucleotide position.

Supplementary Table S1: saltwater crocodilepox virus (SwCRV) genome annotations and comparative analysis of ORFs relative to CRV genome

SwCRV1 synteny	SwCRV2 synteny	CRV synteny	Best PSI-BLASTP hits	SwCRV1 PI %	SwCRV 2 PI %	SwCRV1 AA size	SwCRV2 AA size	CRV AA size	Orthologs, comments, notes
SwCRV1-001	SwCRV2-001	CRV001	hypothetical protein	62.8	62.8	71	71	78	CRV-ORF001
SwCRV1-002	SwCRV2-002		hypothetical protein			238	303		unique to SwCRV-1 and -2, 2 transmembrane helices*
SwCRV1-003	SwCRV2-003		hypothetical protein			117	101		unique to SwCRV-1 and -2, 1 transmembrane helix*
SwCRV1-004	SwCRV2-004	CRV002	hypothetical protein	64.0	63.6	698	698	760	CRV-ORF002
SwCRV1-005	SwCRV2-005		hypothetical protein			75	75		unique to SwCRV-1 and -2
SwCRV1-006	SwCRV2-006	CRV003	hypothetical protein	84.8	84.8	334	334	329	CRV-ORF003
SwCRV1-007	SwCRV2-007	CRV004	hypothetical protein	81.7	81.9	473	473	484	CRV-ORF004
SwCRV1-008	SwCRV2-008	CRV005	hypothetical protein	80.7	80.7	242	242	242	CRV-ORF005
SwCRV1-009	SwCRV2-009	CRV006	hypothetical protein	63.2	63.2	67	67	68	CRV-ORF006
SwCRV1-010	SwCRV2-010	CRV007	hypothetical protein	79.1	79.1	67	67	67	CRV-ORF007
SwCRV1-011	SwCRV2-011	CRV008	hypothetical protein	72.1	72.1	537	537	498	CRV-ORF008
SwCRV1-012	SwCRV2-012	CRV009	F-box domain protein	87.8	87.8	189	189	189	CRV-ORF009
SwCRV1-013	SwCRV2-013	CRV010	F-box domain protein	77.7	77.7	288	288	276	CRV-ORF010
SwCRV1-014	SwCRV2-014	CRV011	F-box domain protein	83.5	83.5	244	244	265	CRV-ORF011
SwCRV1-015	SwCRV2-015	CRV012	F-box domain protein	96.9	96.9	196	196	196	CRV-ORF012
SwCRV1-016	SwCRV2-016		hypothetical protein			304	304		unique to SwCRV-1 and -2
SwCRV1-017	SwCRV2-017	CRV013	F-box domain protein	93.5	93.5	230	230	225	CRV-ORF013
SwCRV1-018	SwCRV2-018	CRV014	F-box domain protein	91.5	91.5	200	200	196	CRV-ORF014
SwCRV1-019	SwCRV2-019	CRV015	F-box domain protein	90.5	90.5	196	205	206	CRV-ORF015
SwCRV1-020	SwCRV2-020		hypothetical protein			132	132		unique to SwCRV-1 and -2
SwCRV1-021	SwCRV2-021	CRV016	hypothetical protein	75.8	75.8	426	439	444	CRV-ORF016
SwCRV1-022	SwCRV2-022	CRV017	hypothetical protein	83.6	83.6	171	171	170	CRV-ORF017
SwCRV1-023	SwCRV2-023	CRV018	hypothetical protein	69.8	69.8	178	178	172	CRV-ORF018

SwCRV1 synteny	SwCRV2 synteny	CRV synteny	Best PSI-BLASTP hits	SwCRV1 PI %	SwCRV 2 PI %	SwCRV1 AA size	SwCRV2 AA size	CRV AA size	Orthologs, comments, notes
SwCRV1-024	SwCRV2-024		hypothetical protein			94	94		unique to SwCRV-1 and -2
SwCRV1-025	SwCRV2-025	CRV019	hypothetical protein	89.5	89.5	159	159	162	CRV-ORF019
SwCRV1-026	SwCRV2-026		hypothetical protein			126	126		unique to SwCRV-1 and -2
SwCRV1-027	SwCRV2-027	CRV020	hypothetical protein	88.9	88.9	135	135	135	CRV-ORF020
SwCRV1-028	SwCRV2-028	CRV021	SORF2 domain protein	90	90	442	442	438	CRV-ORF021
SwCRV1-029	SwCRV2-029		hypothetical protein			217	217		unique to SwCRV-1 and -2
SwCRV1-030	SwCRV2-030	CRV022	hypothetical protein	94.7	94.7	301	301	302	CRV-ORF022
SwCRV1-031	SwCRV2-031	CRV023	hypothetical protein	79.7	79.7	271	271	305	CRV-ORF023
SwCRV1-032	SwCRV2-032	CRV025	hypothetical protein	96.7	96.7	123	123	123	CRV-ORF025
SwCRV1-033	SwCRV2-033	CRV026	F-box domain protein	81.7	81.7	233	233	251	CRV-ORF026
SwCRV1-034	SwCRV2-034	CRV027	hypothetical protein	98.8	98.8	248	248	248	CRV-ORF027
SwCRV1-035	SwCRV2-035		hypothetical protein			81	81		unique to SwCRV-1 and -2
SwCRV1-036	SwCRV2-036		hypothetical protein			70	70		unique to SwCRV-1 and -2
SwCRV1-037	SwCRV2-037	CRV028	hypothetical protein	77.3	77.3	311	311	316	CRV-ORF028
SwCRV1-038	SwCRV2-038	CRV030	J-domain protein	85.5	85.5	237	237	229	CRV-ORF030
SwCRV1-039	SwCRV2-039	CRV031	hypothetical protein	97	97	66	66	68	CRV-ORF031
SwCRV1-040	SwCRV2-040	CRV032	hypothetical protein	92.8	92.8	248	248	250	CRV-ORF032
SwCRV1-041	SwCRV2-041		hypothetical protein			86	86		unique to SwCRV-1 and -2
SwCRV1-042	SwCRV2-042	CRV033	hypothetical protein	75.8	76.5	438	438	476	CRV-ORF033
SwCRV1-043	SwCRV2-043	CRV034	hypothetical protein	62.8	54	612	284	688	CRV-ORF034
SwCRV1-044			hypothetical protein			65			unique to SwCRV-1
	SwCRV2-044	CRV036	hypothetical protein		88		410	213	CRV-ORF036
SwCRV1-045		CRV035	hypothetical protein	78.7		551		548	CRV-ORF035
SwCRV1-046		CRV036	hypothetical protein	89.7		214		213	CRV-ORF036

SwCRV1 synteny	SwCRV2 synteny	CRV synteny	Best PSI-BLASTP hits	SwCRV1 PI %	SwCRV 2 PI %	SwCRV1 AA size	SwCRV2 AA size	CRV AA size	Orthologs, comments, notes
SwCRV1-047	SwCRV2-045	CRV037	serine/threonine protein kinase	97.2	97.2	458	458	457	CRV-ORF037
SwCRV1-048	SwCRV2-046	CRV038	IEV protein	92.9	92.7	683	683	688	CRV-ORF038
SwCRV1-049	SwCRV2-047	CRV039	palmitylated EEV envelope lipase	98.7	98.7	371	371	371	CRV-ORF039
SwCRV1-050	SwCRV2-048		hypothetical protein			57	57		unique to SwCRV-1 and -2
SwCRV1-051	SwCRV2-049	CRV040	B22R-like protein	84.7	84.8	1853	1853	1908	CRV-ORF040
SwCRV1-052	SwCRV2-050	CRV041	B22R-like protein	80	80	1880	1880		CRV-ORF041, fragmented
SwCRV1-053	SwCRV2-051	CRV041	B22R-like protein	67.2	67.1	1385	1385	1874	CRV-ORF041, fragmented
SwCRV1-054	SwCRV2-052		hypothetical protein			71	89		unique to SwCRV-1 and -2
SwCRV1-055	SwCRV2-053	CRV043	B22R-like protein	86.2	86.3	1894	1893	1941	CRV-ORF042
SwCRV1-056	SwCRV2-054	CRV042	RING-like motif protein	84.3	84.3	490	490	555	CRV-ORF043
SwCRV1-057	SwCRV2-055	CRV044	hypothetical protein	86.4	86.4	81	81	81	CRV-ORF044
SwCRV1-058	SwCRV2-056		hypothetical protein			294	294		unique to SwCRV-1 and -2
SwCRV1-059	SwCRV2-057	CRV045	RING-like motif protein	95.1	95.1	603	603	604	CRV-ORF045
SwCRV1-060	SwCRV2-058	CRV046	hypothetical protein	88.5	88.1	215	215	215	CRV-ORF046
SwCRV1-061	SwCRV2-059	CRV047	Apc11-like protein	92.6	93.8	79	79	81	CRV-ORF047
SwCRV1-062	SwCRV2-060	CRV048	hypothetical protein	94.7	94.7	643	643	643	CRV-ORF048
SwCRV1-063	SwCRV2-061		hypothetical protein			660	660		unique to SwCRV-1 and -2
SwCRV1-064	SwCRV2-062	CRV049	hypothetical protein	89.4	89.8	473	476	482	CRV-ORF049
SwCRV1-065	SwCRV2-063	CRV050	hypothetical protein	88.1	88.1	243	243	241	CRV-ORF050
SwCRV1-066	SwCRV2-064	CRV051	site-specific recombinase-like protein	99.6	99.6	229	229	229	CRV-ORF051
SwCRV1-067	SwCRV2-065	CRV052	NAD+-dependent DNA ligase	78.1	78.3	322	322	360	CRV-ORF052
SwCRV1-068	SwCRV2-066		hypothetical protein			531	531		unique to SwCRV-1 and -2
SwCRV1-069	SwCRV2-067		hypothetical protein			159	159		unique to SwCRV-1 and -2

SwCRV1 synteny	SwCRV2 synteny	CRV synteny	Best PSI-BLASTP hits	SwCRV1 PI %	SwCRV 2 PI %	SwCRV1 AA size	SwCRV2 AA size	CRV AA size	Orthologs, comments, notes
SwCRV1-070	SwCRV2-068	CRV053	DNA-binding virion core protein	85.5	85.5	110	110	117	CRV-ORF053
SwCRV1-071	SwCRV2-069	CRV054	poly(A) polymerase large subunit	99.2	99.2	473	473	473	CRV-ORF054
SwCRV1-072	SwCRV2-070		hypothetical protein			492	492		unique to SwCRV-1 and -2
SwCRV1-073	SwCRV2-071	CRV055	E2L/O1L-like protein	88.7	89	776	776	777	CRV-ORF055
SwCRV1-074	SwCRV2-072		hypothetical protein			218	218		unique to SwCRV-1 and -2
SwCRV1-075	SwCRV2-073		hypothetical protein			219	219		unique to SwCRV-1 and -2
SwCRV1-076	SwCRV2-074	CRV056	RNA polymerase subunit RP030	99.5	99.5	188	188	188	CRV-ORF056
SwCRV1-077	SwCRV2-075	CRV057	hypothetical protein	98.8	98.8	566	566	566	CRV-ORF057
SwCRV1-078	SwCRV2-076	CRV058	virion core protein	97	75	570	570		CRV-ORF058, fragmented
SwCRV1-079	SwCRV2-077	CRV058	virion core protein	51.4	51.4	496	496	794	CRV-ORF058, fragmented
SwCRV1-080	SwCRV2-078	CRV059	DNA polymerase	96.7	96.7	989	989	988	CRV-ORF059
SwCRV1-081	SwCRV2-079	CRV060	IMV redox protein	99	99	97	97	97	CRV-ORF060
SwCRV1-082	SwCRV2-080	CRV061	virion core protein	96.9	96.9	128	128	128	CRV-ORF061
SwCRV1-083	SwCRV2-081	CRV062	E2L/OIL-like protein	92.6	92.6	729	729	727	CRV-ORF062
SwCRV1-084	SwCRV2-082	CRV063	E2L/OIL-like protein	95.7	95.7	766	766	765	CRV-ORF063
SwCRV1-085	SwCRV2-083		hypothetical protein			546	546		unique to SwCRV-1 and -2
SwCRV1-086	SwCRV2-084	CRV064	E2L/OIL-like protein	93.2	93.2	755	755	755	CRV-ORF064
SwCRV1-087	SwCRV2-085	CRV065	DNA-binding virion core protein	99.7	99.7	309	309	309	CRV-ORF065
SwCRV1-088	SwCRV2-086	CRV066	hypothetical protein	93.8	93.8	65	65	65	CRV-ORF066
SwCRV1-089	SwCRV2-087	CRV067	DNA-binding phosphoprotein	96.2	96.2	239	239	240	CRV-ORF067
SwCRV1-090	SwCRV2-088		hypothetical protein			608	608		unique to SwCRV-1 and -2
SwCRV1-091	SwCRV2-089	CRV068	IMV membrane protein	98.7	98.7	79	79	79	CRV-ORF068

SwCRV1 synteny	SwCRV2 synteny	CRV synteny	Best PSI-BLASTP hits	SwCRV1 PI %	SwCRV 2 PI %	SwCRV1 AA size	SwCRV2 AA size	CRV AA size	Orthologs, comments, notes
SwCRV1-092	SwCRV2-090	CRV069	telomere binding protein	97.7	97.7	387	387	388	CRV-ORF069
SwCRV1-093	SwCRV2-091	CRV070	virion core proteinase	98.6	98.6	430	430	431	CRV-ORF070
SwCRV1-094	SwCRV2-092	CRV071	RNA helicase NPH-II	99	99	674	674	674	CRV-ORF071
SwCRV1-095	SwCRV2-093	CRV072	metalloprotease	95.6	95.6	624	624	633	CRV-ORF072
SwCRV1-096	SwCRV2-094	CRV073	hypothetical protein	97.2	97.2	106	106	106	CRV-ORF073
SwCRV1-097	SwCRV2-095	CRV074	transcriptional elongation factor	97	97	234	234	233	CRV-ORF074
SwCRV1-098	SwCRV2-096	CRV075	glutaredoxin 2	94.8	94.8	115	115	116	CRV-ORF075
SwCRV1-099	SwCRV2-097	CRV076	virion core protein	95.6	95.6	453	453	455	CRV-ORF076
SwCRV1-100	SwCRV2-098	CRV077	RNA polymerase subunit RPO7	100	100	63	63	63	CRV-ORF077
SwCRV1-101	SwCRV2-099	CRV078	hypothetical protein			188	188	194	CRV-ORF078
SwCRV1-102	SwCRV2-100	CRV079	virion core protein	96.5	96.5	347	347	344	CRV-ORF079
SwCRV1-103	SwCRV2-101	CRV080	late transcription factor VLTF-1	100	100	258	258	258	CRV-ORF080
SwCRV1-104	SwCRV2-102	CRV081	myristylated protein	93	93	329	329	328	CRV-ORF081
SwCRV1-105	SwCRV2-103	CRV082	myristylated IMV envelope protein	99.2	99.2	251	251	251	CRV-ORF082
SwCRV1-106	SwCRV2-104		hypothetical protein			89	89		unique to SwCRV-1 and -2
SwCRV1-107	SwCRV2-105	CRV083	hypothetical protein	93.3	93.3	89	89	89	CRV-ORF083
SwCRV1-108	SwCRV2-106		hypothetical protein			58	58		unique to SwCRV-1 and -2
SwCRV1-109	SwCRV2-107	CRV084	GyrB-like ATPase domain protein	84.7	84.7	467	467	494	CRV-ORF084
SwCRV1-110	SwCRV2-108	CRV085	GyrB-like ATPase domain protein	84.4	84.4	462	462	481	CRV-ORF085
SwCRV1-111	SwCRV2-109		hypothetical protein			422	422		unique to SwCRV-1 and -2
SwCRV1-112	SwCRV2-110	CRV086	GyrB-like ATPase domain protein	87.1	87.1	393	393	392	CRV-ORF086
SwCRV1-113	SwCRV2-111		hypothetical protein			57	57		unique to SwCRV-1 and -2

SwCRV1 synteny	SwCRV2 synteny	CRV synteny	Best PSI-BLASTP hits	SwCRV1 PI %	SwCRV 2 PI %	SwCRV1 AA size	SwCRV2 AA size	CRV AA size	Orthologs, comments, notes
SwCRV1-114	SwCRV2-112	CRV087	GyrB-like ATPase domain protein	81.3	81.3	366	366	417	CRV-ORF087
SwCRV1-115	SwCRV2-113		hypothetical protein			166	166		unique to SwCRV-1 and -2, 1 transmembrane helix*
SwCRV1-116	SwCRV2-114	CRV088	GyrB-like ATPase domain protein	95.2	95.2	415	415	415	CRV-ORF088
SwCRV1-117	SwCRV2-115	CRV089	GyrB-like ATPase domain protein	84.8	84.8	503	503	509	CRV-ORF089
SwCRV1-118	SwCRV2-116		hypothetical protein			115	115		unique to SwCRV-1 and -2
SwCRV1-119	SwCRV2-117	CRV090	GyrB-like ATPase domain protein	91.2	91.2	1017	1017	1017	CRV-ORF090
SwCRV1-120	SwCRV2-118	CRV091	hypothetical protein	97.9	97.9	97	97	97	CRV-ORF091
SwCRV1-121	SwCRV2-119	CRV092	hypothetical protein	97	97	302	302	302	CRV-ORF092
SwCRV1-122	SwCRV2-120	CRV093	DNA-binding virion core protein	94.3	94.3	245	245	245	CRV-ORF093
SwCRV1-123	SwCRV2-121	CRV094	IMV membrane protein	95.4	95.4	129	129	130	CRV-ORF094
SwCRV1-124	SwCRV2-122	CRV095	IMV membrane protein	98.7	98.7	156	156	156	CRV-ORF095
SwCRV1-125	SwCRV2-123	CRV096	poly(A) polymerase small subunit	98.6	98.6	294	294	294	CRV-ORF096
SwCRV1-126	SwCRV2-124	CRV097	RNA polymerase subunit RPO22	98.9	98.9	189	189	189	CRV-ORF097
SwCRV1-127	SwCRV2-125	CRV098	hypothetical protein	97.7	97.7	132	132	132	CRV-ORF098
SwCRV1-128	SwCRV2-126	CRV099	RNA polymerase subunit RPO147	99.5	99.5	1289	1289	1289	CRV-ORF099
SwCRV1-129	SwCRV2-127	CRV100	hypothetical protein	97.3	97.3	75	75	75	CRV-ORF100
SwCRV1-130	SwCRV2-128	CRV101	tyrosine|serine protein posphatase	98.8	98.8	167	167	167	CRV-ORF101
SwCRV1-131	SwCRV2-129	CRV102	IMV membrane protein	96.1	96.1	233	233	233	CRV-ORF102
SwCRV1-132	SwCRV2-130	CRV103	IMV envelope protein	95.7	95.7	278	278	278	CRV-ORF103
SwCRV1-133	SwCRV2-131	CRV104	RNA-polymerase associated protein	99.1	99.1	800	800	800	CRV-ORF104

SwCRV1 synteny	SwCRV2 synteny	CRV synteny	Best PSI-BLASTP hits	SwCRV1 PI %	SwCRV 2 PI %	SwCRV1 AA size	SwCRV2 AA size	CRV AA size	Orthologs, comments, notes
SwCRV1-134	SwCRV2-132		hypothetical protein			85	85		unique to SwCRV-1 and -2
SwCRV1-135	SwCRV2-133	CRV105	late transcription factor VLTF-4	94.3	94.3	123	123	123	CRV-ORF105
SwCRV1-136	SwCRV2-134	CRV106	DNA topoisomerase IB	98.5	98.8	324	324	324	CRV-ORF106
SwCRV1-137	SwCRV2-135	CRV107	hypothetical protein	95.8	95.8	142	142	142	CRV-ORF107
SwCRV1-138	SwCRV2-136	CRV108	hypothetical protein	95.8	95.8	119	119	119	CRV-ORF108
SwCRV1-139	SwCRV2-137	CRV109	mRNA capping enzyme large subunit	98.2	98.2	848	848	848	CRV-ORF109
SwCRV1-140	SwCRV2-138	CRV110	virion core protein	90.8	90.8	118	118	118	CRV-ORF110
SwCRV1-141	SwCRV2-139	CRV111	virion core protein	87.1	87.7	302	300	292	CRV-ORF111
SwCRV1-142	SwCRV2-140		hypothetical protein			272	270		unique to SwCRV-1 and -2
SwCRV1-143	SwCRV2-141	CRV112	uracil DNA glycosylase	98.2	98.2	225	225	225	CRV-ORF112
SwCRV1-144	SwCRV2-142		hypothetical protein			151	151		unique to SwCRV-1 and -2
SwCRV1-145	SwCRV2-143	CRV113	NTPase	98.9	98.9	782	782	781	CRV-ORF113
	SwCRV2-144		early transcription factor VETFs	50			80		ORFV PP69
SwCRV1-146			virion protein	50		731			ORFV PP66
SwCRV1-147			early transcription factor VETFs	51		80			ORFV PP69
SwCRV1-148	SwCRV2-145	CRV114	early transcription factor small subunit	99.7	99.7	633	633	633	CRV-ORF114
	SwCRV2-146		virion protein				213		ORFV PP66
SwCRV1-149	SwCRV2-147	CRV115	RNA polymerase subunit RPO18	98.8	98.8	165	165	165	CRV-ORF115
SwCRV1-150	SwCRV2-148		hypothetical protein			138	138		unique to SwCRV-1 and -2
SwCRV1-151	SwCRV2-149	CRV116	MutT motif protein	92.7	92.7	245	245	232	CRV-ORF116
SwCRV1-152	SwCRV2-150	CRV117	MutT motif protein	88.8	88.8	233	233	250	CRV-ORF117
SwCRV1-153	SwCRV2-151	CRV118	NPH-1	98.4	98.4	625	625	626	CRV-ORF118

SwCRV1 synteny	SwCRV2 synteny	CRV synteny	Best PSI-BLASTP hits	SwCRV1 PI %	SwCRV 2 PI %	SwCRV1 AA size	SwCRV2 AA size	CRV AA size	Orthologs, comments, notes
SwCRV1-154	SwCRV2-152	CRV119	mRNA capping enzyme small subunit	98.3	97.9	289	289	289	CRV-ORF119
SwCRV1-155	SwCRV2-153		hypothetical protein			282	271		unique to SwCRV-1 and -2
SwCRV1-156			hypothetical protein			551			ORFV PP219
SwCRV1-157	SwCRV2-154	CRV120	rifampin resistance protein	99.3	99.3	544	544	544	CRV-ORF120
SwCRV1-158	SwCRV2-155	CRV121	late transcription factor VLTF-2	97.9	97.9	146	146	146	CRV-ORF121
SwCRV1-159	SwCRV2-156		hypothetical protein			147	147		unique to SwCRV-1 and -2
SwCRV1-160	SwCRV2-157	CRV122	late transcription factor VLTF-3	99.1	99.1	225	225	225	CRV-ORF122
SwCRV1-161	SwCRV2-158	CRV123	virion redox protein	100	100	69	69	69	CRV-ORF123
SwCRV1-162	SwCRV2-159	CRV124	virion core protein P4b	97.8	97.6	673	672	675	CRV-ORF124
SwCRV1-163	SwCRV2-160	CRV125	virion core protein	87.8	87.8	88	88	90	CRV-ORF125
SwCRV1-164	SwCRV2-161	CRV126	RNA polymerase subunit RPO19	99.4	98.7	158	158	158	CRV-ORF126
SwCRV1-165	SwCRV2-162	CRV127	hypothetical protein	89.5	89.5	520	520	530	CRV-ORF127
SwCRV1-166	SwCRV2-163	CRV128	early transcription factor large subunit	99.4	99.4	699	699	699	CRV-ORF128
SwCRV1-167	SwCRV2-164	CRV129	intermediate transcription factor VITF-3	99	99	292	292	292	CRV-ORF129
SwCRV1-168	SwCRV2-165	CRV130	IMV membrane protein	100	100	74	74	74	CRV-ORF130
SwCRV1-169	SwCRV2-166		hypothetical protein			876	876		unique to SwCRV-1 and -2
SwCRV1-170	SwCRV2-167	CRV131	virion core protein P4a	97.5	97.5	903	903	908	CRV-ORF131
SwCRV1-171	SwCRV2-168	CRV132	nonstructural protein	89.5	89.5	296	296	315	CRV-ORF132
SwCRV1-172	SwCRV2-169	CRV133	virion core protein	94.3	94.3	137	137	140	CRV-ORF133
SwCRV1-173	SwCRV2-170	CRV134	hypothetical protein	76.4	76.4	113	113	127	CRV-ORF134
SwCRV1-174	SwCRV2-171	CRV135	IMV membrane protein	91	91	65	65	67	CRV-ORF135
SwCRV1-175	SwCRV2-172	CRV136	IMV membrane protein	96.9	96.9	96	96	96	CRV-ORF136

**SwCRV1 synteny**	**SwCRV2 synteny**	**CRV synteny**	**Best PSI-BLASTP hits**	**SwCRV1 PI %**	**SwCRV 2 PI %**	**SwCRV1 AA size**	**SwCRV2 AA size**	**CRV AA size**	**Orthologs, comments, notes**
SwCRV1-176	SwCRV2-173		hypothetical protein			152	152		unique to SwCRV-1 and -2
SwCRV1-177	SwCRV2-174	CRV137	IMV membrane protein	98.1	98.1	53	53	53	CRV-ORF137
SwCRV1-178	SwCRV2-175	CRV138	virion core protein	94.7	94.7	95	95	95	CRV-ORF138
SwCRV1-179	SwCRV2-176	CRV139	Myristylated membrane protein	93.6	93.6	388	388	388	CRV-ORF139
SwCRV1-180	SwCRV2-177	CRV140	IMV membrane protein	98.3	98.3	175	175	175	CRV-ORF140
SwCRV1-181	SwCRV2-178	CRV141	DNA helicase	99.6	99.6	459	459	459	CRV-ORF141
SwCRV1-182	SwCRV2-179	CRV142	hypothetical protein	88.6	88.6	87	87	88	CRV-ORF142
SwCRV1-183	SwCRV2-180	CRV143	IMV membrane protein	98.1	98.1	104	104	104	CRV-ORF143
SwCRV1-184	SwCRV2-181	CRV144	DNA polymerase processivity factor	96.2	96.4	444	444	444	CRV-ORF144
SwCRV1-185	SwCRV2-182	CRV145	holliday junction resolvase	91.9	91.9	156	156	160	CRV-ORF145
SwCRV1-186	SwCRV2-183	CRV146	intermediate transcription factor VITF-3	96.1	96.1	379	379	383	CRV-ORF146
SwCRV1-187	SwCRV2-184	CRV147	RNA polymerase subunit RPO132	99.8	99.8	1149	1149	1149	CRV-ORF147
SwCRV1-188	SwCRV2-185	CRV148	IMV A type inclusion-like protein P4c	86.3	62.3	444	318	446	CRV-ORF148
SwCRV1-189	SwCRV2-186	CRV149	IMV membrane protein	98.6	97.8	139	139	139	CRV-ORF149
SwCRV1-190	SwCRV2-187	CRV150	RNA polymerase subunit RPO35	98	98	303	303	303	CRV-ORF150
SwCRV1-191	SwCRV2-188	CRV151	virion core protein	95.8	95.8	71	71	71	CRV-ORF151
SwCRV1-192	SwCRV2-189	CRV152	hypothetical protein	97.3	97.3	111	111	111	CRV-ORF152
SwCRV1-193	SwCRV2-190		hypothetical protein			117	117		unique to SwCRV-1 and -2
SwCRV1-194	SwCRV2-191	CRV153	hypothetical protein	97.5	97.5	119	119	119	CRV-ORF153
SwCRV1-195	SwCRV2-192	CRV154	ATPase	98.8	98.8	249	249	249	CRV-ORF154
SwCRV1-196	SwCRV2-193		hypothetical protein			212	212		unique to SwCRV-1 and -2
SwCRV1-197	SwCRV2-194	CRV155	hypothetical protein	96.9	96.9	226	226	226	CRV-ORF155

SwCRV1 synteny	SwCRV2 synteny	CRV synteny	Best PSI-BLASTP hits	SwCRV1 PI %	SwCRV 2 PI %	SwCRV1 AA size	SwCRV2 AA size	CRV AA size	Orthologs, comments, notes
SwCRV1-198	SwCRV2-195	CRV157	hypothetical protein	89.8	89	127	127	127	CRV-ORF157
SwCRV1-199	SwCRV2-196	CRV158	EEV envelope protein	95.1	95.1	162	162	162	CRV-ORF158
SwCRV1-200	SwCRV2-197	CRV159	hypothetical protein	90.7	91.7	108	108	108	CRV-ORF159
SwCRV1-201	SwCRV2-198	CRV160	hypothetical protein	79.5	78.7	244	244	250	CRV-ORF160
SwCRV1-202	SwCRV2-199	CRV161	hypothetical protein	88	88	132	132	133	CRV-ORF161
SwCRV1-203	SwCRV2-200	CRV162	hypothetical protein	86.9	85.9	99	99	96	CRV-ORF162
SwCRV1-204	SwCRV2-201	CRV163	hypothetical protein	72.4	72.2	460	460	477	CRV-ORF163
SwCRV1-205	SwCRV2-202		hypothetical protein			50	50		unique to SwCRV-1 and -2
SwCRV1-206	SwCRV2-203	CRV164	hypothetical protein	69	68.6	115	118	111	CRV-ORF164
SwCRV1-207	SwCRV2-204		hypothetical protein			54	54		unique to SwCRV-1 and -2
SwCRV1-208	SwCRV2-205	CRV165	KDEL motif protein	77.4	77	243	243	234	CRV-ORF165
SwCRV1-209	SwCRV2-206	CRV166	hypothetical protein	81.7	81.7	188	188	189	CRV-ORF166
SwCRV1-210	SwCRV2-207		hypothetical protein			70	70		unique to SwCRV-1 and -2
SwCRV1-211	SwCRV2-208	CRV167	F-box domain protein	77	76.6	263	263	273	CRV-ORF167
SwCRV1-212	SwCRV2-209	CRV168	hypothetical protein	68.1	68.5	623	623	645	CRV-ORF168
SwCRV1-213	SwCRV2-210	CRV169	hypothetical protein	70.7	70.7	170	170	177	CRV-ORF169
SwCRV1-214	SwCRV2-211		hypothetical protein			60	60		unique to SwCRV-1 and -2
SwCRV1-215	SwCRV2-212	CRV170	hypothetical protein	80.9	80.9	196	196	199	CRV-ORF170
SwCRV1-216	SwCRV2-213		hypothetical protein			285	192		unique to SwCRV-1 and -2
SwCRV1-217	SwCRV2-214		hypothetical protein			238	230		unique to SwCRV-1 and -2
SwCRV1-218	SwCRV2-215	CRV173	hypothetical protein	62.8	62.8	71	71	78	CRV-ORF173

The percent identity and alignment length are taken directly from BLASTP searches. The abbreviations for poxviruses were used: ORFV, Orf virus; CRV; Nile crocodilepox virus; SwCRV-1, saltwater crocodilepox virus subtype 1; SwCRV-2, Saltwater crocodilepox virus subtype 2. Grey shading highlighted box represents the translocated genes between SwCRV and CRV genomes.
*Transmembrane helices are predicted by HHpred software package using default parameters.
